# Complete Genome Sequence of *Desulfuromonas* sp. Strain AOP6, an Iron(III) Reducer Isolated from Subseafloor Sediment

**DOI:** 10.1128/MRA.01325-19

**Published:** 2020-03-19

**Authors:** Yong Guo, Tomo Aoyagi, Tomohiro Inaba, Yuya Sato, Hiroshi Habe, Tomoyuki Hori

**Affiliations:** a Environmental Management Research Institute, National Institute of Advanced Industrial Science and Technology (AIST), Tsukuba, Ibaraki, Japan; University of Southern California

## Abstract

*Desulfuromonas* sp. strain AOP6, with iron(III)-reducing activity, was isolated from subseafloor sediment in Nankai Trough. We report the complete genome of this strain determined by Illumina MiSeq sequencing and PCR/Sanger sequencing-based gap closing. The genome includes the genes encoding *c*-type cytochromes, type IV pili, and fatty acid degradation enzymes.

## ANNOUNCEMENT

Microbial dissimilatory iron(III) reduction is an important anaerobic respiration process (1–4). Some iron(III)-reducing bacteria, such as *Geobacter* spp., can reduce not only soluble ferric iron but also poorly crystalline Fe(III) oxides (1, 5). However, the mechanisms underlying electron transfer from microorganisms to crystalline Fe(III) oxides are largely unknown, partially because of the limited number of available genome sequences. In our previous work, *Desulfuromonas* sp. strain AOP6 (formerly described as *Pelobacter* sp. AOP6) was isolated from subseafloor sediment in Nankai Trough using acetate and goethite (α-FeOOH) as the electron donor and acceptor, respectively (6). The phylogenetically closest relative of this strain was Desulfuromonas palmitatis (7), followed by Pelobacter acetylenicus (8) and Geoalkalibacter subterraneus (9). Here, we determined the complete genome sequence of the strain by a two-step assembly of the paired-end and mate pair reads. The gaps were further closed by PCR and Sanger sequencing.

Strain AOP6 was anaerobically grown in a modified Widdel medium containing acetate and ferric nitrilotriacetic acid [Fe(III)-NTA] as the electron donor and acceptor, respectively (6). The genomic DNA was extracted by a phenol extraction method, including chemical cell lysis. A paired-end library (insert size, ∼500 bp) and a mate pair library (insert size, ∼4,000 bp) were prepared using a NEBNext Ultra DNA library prep kit for Illumina (New England BioLabs, Ipswich, MA, USA) and a Nextera mate pair sample prep kit (Illumina, San Diego, CA, USA), respectively (10). These two libraries were sequenced with the Illumina MiSeq platform. The paired-end and mate pair libraries yielded 1,965,922 and 897,788 250-bp paired-end reads, respectively. After a preassembly read quality check using Sickle v1.33 (https://github.com/najoshi/sickle/releases/tag/v1.33) with default settings, a two-step combination of the genome assembly was performed. Briefly, the obtained sequence was preassembled using Unicycler v0.4.8 (11) with default settings and then assembled using SPAdes v3.13.0 (12) with the option “trusted-contigs” (13). This resulted in one scaffold with a total size of 3.27 Mbp. To obtain a complete genome sequence, the gaps within the scaffold were closed by sequencing the PCR-amplified DNA fragments with a 3730xl DNA analyzer (Applied Biosystems, Thermo Fisher Scientific, Waltham, MA, USA). Detailed information about the PCR primer and conditions is provided at https://doi.org/10.6084/m9.figshare.11912058.v1 and https://doi.org/10.6084/m9.figshare.11912082.v1.

**FIG 1:**
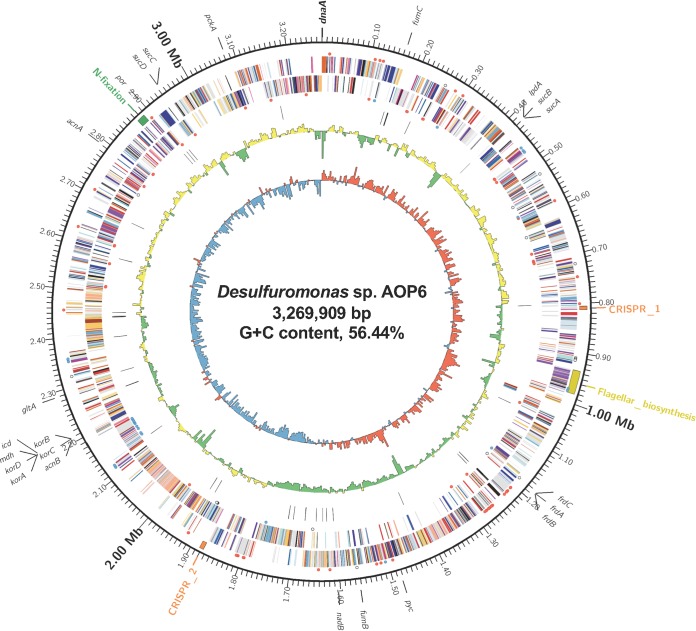
Schematic spherical illustration of the complete genome of *Desulfuromonas* sp. strain AOP6. Ring 1, GC skew at 5-kb windows (red, >0; blue, <0); ring 2, GC content at 5-kb windows (yellow, >56.44%; green, <56.44%); ring 3, rRNA operons (red and blue) and tRNA loci (black); rings 4 and 5, predicted CDSs transcribed in a counterclockwise/clockwise direction and colored by COG category; ring 6, CRISPR (orange), nitrogen fixating (green), and flagellar biosynthesis (yellow) gene clusters; ring 7, scale in megabases. The red and blue circles next to rings 4 and 5 indicate the genes encoding *c*-type cytochromes and type IV pili, respectively, and the gray circles indicate the genes responsible for fatty acid degradation. The positions of the *dnaA*-encoding chromosomal replication initiator (bold italic) and the genes involved in the tricarboxylic acid (TCA) cycle (italic) are shown around the scale ring.

The complete genome sequence of strain AOP6 consists of a 3,269,909-bp circular chromosome (GC content, 56.44%; coverage, 191×). Two *rrn* operons, 2 CRISPR features, 50 tRNA loci, and 3,000 protein-coding sequences (CDSs) were predicted using the DDBJ Fast Annotation and Submission Tool (DFAST) v1.2.2 (14) with default settings except for using Prodigal v2.6.3 for gene identification (Fig. 1). Functional predictions of CDSs using the DFAST default reference database v1.1.5, TIGRFAMs v15.0, and the NCBI Clusters of Orthologous Groups of proteins (COG) 2003–2014 database showed that the genome of strain AOP6 possessed 31 and 71 CDSs for type IV pili and *c*-type cytochrome biosynthesis, respectively. Some *Geobacter* spp. can produce type IV pili that directly transport electrons to crystalline Fe(III) oxides (15, 16). Moreover, the genome possessed 20 genes responsible for fatty acid degradation, which was expected to be associated with Fe(III) reduction (7). Additionally, the complete gene set for the tricarboxylic acid (TCA) cycle, as well as gene clusters for flagellar biosynthesis and nitrogen fixation, were found in the genome. The information gleaned from the complete genome reveals the ability of strain AOP6 to transfer electrons to Fe(III), which furthers our understanding of *Desulfuromonas* species-driven, carbon, and nitrogen transformation in natural environments.

### Data availability.

The complete genome sequence of *Desulfuromonas* sp. strain AOP6 has been deposited in DDBJ/ENA/GenBank under accession number AP022810. The raw sequence reads for the paired-end and mate pair libraries are available at accession numbers DRR194005 and DRR194004, respectively.
